# Human Naa50 Shows Serotonin *N*-Acetyltransferase Activity, and Its Overexpression Enhances Melatonin Biosynthesis, Resulting in Osmotic Stress Tolerance in Rice

**DOI:** 10.3390/antiox12020319

**Published:** 2023-01-30

**Authors:** Kyungjin Lee, Kyoungwhan Back

**Affiliations:** Department of Biotechnology, College of Agriculture and Life Sciences, Chonnam National University, Gwangju 61186, Republic of Korea

**Keywords:** archaea, human, Naa50, *N*-acetylserotonin, synthetic gene, melatonin, transgenic rice

## Abstract

A new clade of serotonin *N*-acetyltransferase (SNAT), the penultimate enzyme in the melatonin biosynthetic pathway, has been reported in the archaeon *Thermoplasma volcanium*. The closest homolog of archaea SNAT in human was an *N*-alpha-acetyltransferase50 (Naa50). To determine whether human Naa50 (hNaa50) shows SNAT enzyme activity, we chemically synthesized and expressed the *hNaa50* gene in *Escherichia coli*, followed by Ni^2+^ affinity purification. Purified recombinant hNaa50 showed SNAT activity (*K*_m_ and *V*_max_ values of 986 μM and 1800 pmol/min/mg protein, respectively). To assess its in vivo function, *hNaa50* was overexpressed in rice (*hNaa50-*OE). The transgenic rice plants produced more melatonin than nontransgenic wild-type rice, indicating that *hNaa50* is functionally coupled with melatonin biosynthesis. Due to its overproduction of melatonin, *hNaa50*-OE had a higher tolerance against osmotic stress than the wild type. Enhanced expression of the chaperone genes *BIP1* and *CNX* in *hNaa50*-OE plants was responsible for the increased tolerance. It is concluded that *hNaa50* harbors serotonin *N*-acetyltransferase enzyme activity in addition to its initial *N*-alpha-acetyltransferase, suggesting the bifunctionality of the *hNaa50* enzyme toward serotonin and protein substrates. Consequently, ectopic overexpression of *hNaa50* in rice enhanced melatonin synthesis, indicating that *hNaa50* is in fact involved in melatonin biosynthesis.

## 1. Introduction

Serotonin *N*-acetyltransferase (SNAT.; also named arylakylamine *N*-acetyltransferase) is the penultimate or final enzyme of melatonin biosynthesis, depending on the substrate, in both animals and plants [1,2]. SNAT catalyzes the conversion of serotonin and 5-methoxytryptamine into *N*-acetylserotonin and melatonin, respectively. In humans, *SNAT* exists as a single copy and plays an important role in melatonin synthesis, which is increased in darkness by enhanced expression of SNAT activity. This results in a daily rhythm of melatonin, which regulates the biological clock in vertebrates [3]. Melatonin is a potent antioxidant that not only scavenges up to 10 molecules of reactive oxygen species or reactive nitrogen species [4], but also induces a number of antioxidant enzymes [1,5]. In humans, *SNAT* is expressed preferentially in the pineal gland and retina; it has not been detected in other tissues such as the heart, liver, and cortex [6]. All tissues containing mitochondria can produce melatonin [7,8]. However, *SNAT* mRNA is absent in many human melatonin-synthesizing tissues, which is indicative of the presence of other *SNAT* genes [9].

A novel *SNAT* has been cloned and enzymatically characterized from the archaeon *Thermoplasma volcanium* (TvSNAT) [10]. TvSNAT exhibits *N*-acetyltransferase activity to a wide range of substrates, including tyramine, tryptamine, serotonin, 5-methoxytryptamine, octopamine, and spermidine. It does not show amino acid sequence homology to reported SNAT proteins from animals or plants. The gene with the highest sequence homology to TvSNAT in animals and plants is *N*-alpha-acetyltransferase50 (Naa50 or NatE). Naa50 is an *N*-alpha-acetyltransferase (NAT) that transfers an acetyl group from acetyl conenzyme A to the α-amino group of the first residue of proteins. In cells, N-terminal acetylation by NATs is important for regulating protein stability, protein–protein interactions, and endoplasmic reticulum translocation [11].

All SNAT proteins from animals and plants belong to the GCN-related *N*-acetyltransferase (GNAT) superfamily, which transfers an acetyl group to diverse substrates, including proteins and chemicals [12]. Although the GNAT superfamily has a common protein motif, their amino acid sequence homology is extremely low. Moreover, no amino acid identity has been observed between animal and plant SNATs [13], although they share serotonin as a substrate for melatonin synthesis [2]. Interestingly, *Arabidopsis thaliana* SNAT accepts a broad range of substrates such as histones [14], chloroplast proteins [15], and serotonin [16], to which it adds an acetyl group to facilitate virus movement, state transition, and melatonin biosynthesis, respectively.

The aim of this work was to determine whether or not human *Naa50*, the closest homolog gene of archaeal *SNAT*, harbors SNAT enzyme activity in vitro as well as being functionally coupled to melatonin biosynthesis in vivo.

To achieve this goal, we chemically synthesized human *Naa50* (*hNaa50*), an archaeal *SNAT* homolog, expressed in *Escherichia coli*, and purified recombinant hNaa50, followed by in vitro SNAT enzyme kinetic analyses. Furthermore, the in vivo function of hNaa50 for melatonin biosynthesis has been investigated in transgenic rice plants overexpressing *hNaa50*.

## 2. Materials and Methods

### 2.1. Synthesis of the Human Naa50 Gene

Based on the sequence of the human Naa50 protein (GenBank accession number BAB14397), we manually designed the corresponding nucleotide sequence according to rice *SNAT2* codon usage (GenBank accession number AK068156) [17]. The codon-modified *hNaa50* was custom-synthesized at Bioneer (Daejeon, Republic of Korea).

### 2.2. Expression in Escherichia coli and Purification of Recombinant hNaa50 Protein

The full-length synthetic *hNaa50* gene was amplified by PCR (*hNaa50* forward primer, 5′-AAA AAG CAG GCT CCA TGA AGG GCT CGC GCA-3′; *hNaa50* reverse primer, 5′-AGA AAG CTG GGT TCA GTT GTC CGT CTT CTG-3′ with a template plasmid pBHA-hNaa50 containing synthetic *hNaa50* DNA (Bioneer). The first PCR product was used as the template for a second PCR using an *attB* primer set (*attB* forward primer 5′-GGG GAC AAG TTT GTA CAA AAA AGC AGG CT-3′ and *attB* reverse primer 5′-GGG GAC CAC TTT GTA CAA GAA AGC TGG GT-3′). The second *hNaa50* PCR product was cloned via Gateway recombination reactions into the pDONR221 vector (Invitrogen, Carlsbad, CA, USA), and then into the destination vector pET300/NT-DEST (Invitrogen) according to the manufacturer’s procedure. The pET300-hNaa50 plasmid was transformed into *E*. *coli* strain BL21(DE3) (Invitrogen). An overnight culture (10 mL) grown in the presence of the antibiotic ampicillin (50 mg/L) was inoculated into 100 mL Terrific Broth medium (20 g/L Bacto-tryptone, 24 g/L Bacto-yeast extract, glycerol 4 mL/L, and phosphate buffer [0.017 M KH_2_PO_4_ and 0.072 M K_2_HPO_4_]) containing 50 mg/L ampicillin and incubated at 37 °C for 4 h, followed by the addition of 1 mM isopropyl-*β*-D-thiogalactopyranoside (IPTG.; Sigma, St. Louis, MO, USA). The culture was incubated at 28 °C with shaking at 180 rpm for 5 h. Affinity (Ni^2+^) chromatography purification was performed according to the manufacturer’s recommendations (Qiagen, Tokyo, Japan).

### 2.3. Homology Analysis

Analysis of amino acid sequence homology was carried out using the BLASTp tool in the non-redundant protein sequences database of the National Center for Biotechnology Information (http://www.ncbi.nlm.nih.gov/, accessed on 7 November 2019).

### 2.4. Serotonin N-Acetyltransferase Enzyme Kinetics

Purified recombinant hNaa50 protein was incubated in a total volume of 100 µL containing 0.5 mM serotonin and 0.5 mM acetyl-CoA in 100 mM potassium phosphate (pH 7.8 or varying pH) at 45 °C (or other temperatures) for 30 min as described previously [10]. Enzymatic reaction products such as *N*-acetylserotonin (NAS) and melatonin were subjected to high-performance liquid chromatography (HPLC) as described previously [18]. Substrate affinity (*K*_m_) and the maximum reaction rate (*V*_max_) were calculated using Lineweaver–Burk plots. Protein concentrations were determined using the Bradford method and a protein assay dye (Bio-Rad Laboratories Inc., Hercules, CA, USA). The analysis was performed in triplicate.

### 2.5. Subcellular Localization of hNaa50 in Tobacco (Nicotiana benthamiana)

The pER-mCherry binary vector for subcellular localization analysis of hNaa50 was kindly donated by Dr. H.G. Kang (Texas State University, San Marcos, TX, USA). Full-length synthetic *hNaa50* DNA was amplified via PCR using a primer set containing *Asc*I sites (*Asc*I forward primer: 5′-GGC GCG CCA TGA AGG GCT CGC GCA TC-3′; *Asc*I reverse primer: 5′-GGC GCG CCG GTT GTC CGT CTT CTG GAC-3′) with plasmid pBHA-hNaa50 as the template. The resulting *hNaa50* PCR product was cloned into the TA vector (RBC Bioscience, New Taipei City, Taiwan), followed by *Asc*I digestion. The *Asc*I insert of *hNaa50* was ligated into the *Asc*I site of the binary vector pER8-mCherry containing the estrogen-inducible XVE promoter (Pxve), resulting in pER8-hNaa50-mCherry. The pER8-hNaa50-mCherry plasmid was transferred into *Agrobacterium tumefaciens* strain GV2260 using the freeze-thaw method. *Agrobacterium*-mediated transient expression of hNaa50-mCherry fusion protein and confocal microscopy (TCS-SP5; Leica, Wetzlar, Germany) were previously described [19].

### 2.6. Transgenic Rice Plants Overexpressing hNaa50

For ectopic overexpression of synthetic *hNaa50* under the control of the maize ubiquitin promoter, we employed the pIPKb002 binary vector [20]. The pDONR221-hNaa50 plasmid isolated from *E*. *coli* culture was recombined with the pIPKb002 destination vector by LR (between the *attL* and the *attR* sites) recombination to yield the pIPKb002-hNaa50 binary plasmid. The pIPKb002-hNaa50 binary vector was transformed into *A*. *tumefaciens* LBA4404, followed by transformation into calli derived from the mature seeds of the Korean *japonica* rice cultivar (*Oryza sativa* cv. Dongjin). The transgenic rice plants were regenerated from calli in the presence of hygromycin via a somatic embryogenesis process as described previously [21].

### 2.7. Characterization of hNaa50-Overexpression Transgenic Rice Plants

Homozygous T_2_ transgenic rice seeds were used in further studies. Dehusked wild-type and transgenic rice seeds were sterilized with 2% NaOCl for 50 min, thoroughly rinsed with sterile distilled water, and sown on half-strength Murashige and Skoog (MS) medium under cool daylight fluorescent lamps (60 μmol m^−2^ s^−1^) (Philips, Amsterdam, The Netherlands) under a 14 h light/10 h dark photoperiod at 28 °C/24 °C (day/night). Seven-day-old seedlings were used in further experiments. For mannitol (Sigma-Aldrich, St. Louis, MO, USA) treatment, surface-sterilized rice seeds were sown and grown on half-strength MS medium containing various concentrations of mannitol. Melatonin contents were measured in frozen samples (0.1 g) that were pulverized to a powder in liquid nitrogen using the TissueLyser II (Qiagen, Tokyo, Japan). The sample powders were extracted with 1 mL chloroform, followed by centrifugation for 10 min at 12,000× *g*, and the supernatants (200 µL) were evaporated and dissolved in 0.1 mL 40% methanol. The resulting 10 µL aliquots were subjected to high-performance liquid chromatography (HPLC) with a fluorescence detector system (Waters, Milford, MA, USA) as described previously [19]. Melatonin was eluted after about 31 min under the HPLC conditions. The measurements were performed in triplicate.

### 2.8. Total RNA Isolation and Reverse Transcription–Polymerase Chain Reaction (RT-PCR)

Total RNA was isolated from rice leaves from 7-day-old seedlings grown in MS medium or from MS medium containing 150 mM mannitol using a Ribospin Plant Kit (GeneAll Biotechnology Co., Seoul, Republic of Korea). First-strand cDNA was synthesized from 1 µg total RNA using RevertAid Reverse Transcriptase (Thermo Scientific Fermentas, St. Leon-Rot, Germany) and oligo(dT) primers (Promega, Madison, WI, USA). Thereafter, 0.2 µL of the reverse transcription reaction was used as the template for PCR amplification. We analyzed the expression of stress-related genes by RT-PCR. The rice ubiquitin-5 gene (*UBQ5*) was used as the loading control. The sequences of primers were previously described [22,23,24] (Table S1). The amplified fragments were electrophoresed on ethidium bromide gels and photographed under ultraviolet (UV) light.

### 2.9. Quantitative Real Time (qRT)-PCR Analyses

To quantify the expression levels of genes, real-time PCR (qRT-PCR) was carried out in a Mic qPCR Cycler system (Bio Molecular Systems, Queensland, VIC, Australia) with specific primers and the Luna Universal qPCR Master Mix (New England Biolabs, Ipswich, MA, USA). The expression of genes was calculated using Mic’s RQ software v2.2 (Bio Molecular Systems) and normalized to the rice ubiquitin-5 gene (UBQ5). The primer sequences used in the qRT-PCR analyses are shown in Table S1.

### 2.10. Statistical Analysis

The data were analyzed by analysis of variance using IBM SPSS Statistics 23 software (IBM Corp., Armonk, NY, USA). Means with different letters indicate significantly different values at *p* < 0.05 according to Tukey’s post hoc honestly significant difference (HSD) test. Data are presented as means ± standard deviations.

## 3. Results

### 3.1. Selection and Synthesis of the Human Naa50 Gene

**Figure 1:**
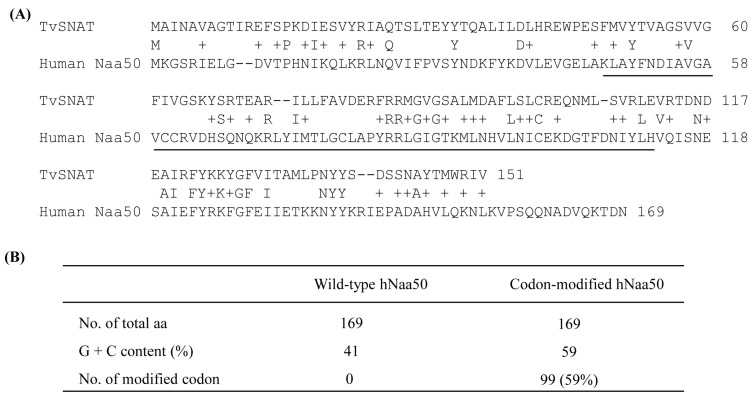
(**A**) Amino acid sequences of *Thermoplasma volcanium* serotonin *N*-acetyltransferase (TvSNAT.; NC_002689) and human (*Homo sapiens*) Naa50 (BAB14397). (**B**) Modification of *hNaa50* codons. The nucleotide sequence of synthetic *hNaa50* was codon-optimized with reference to the rice *SNAT2* codon. The *N*-acetyltransferase domain is underlined. +, similar amino acids.

**Figure 2:**
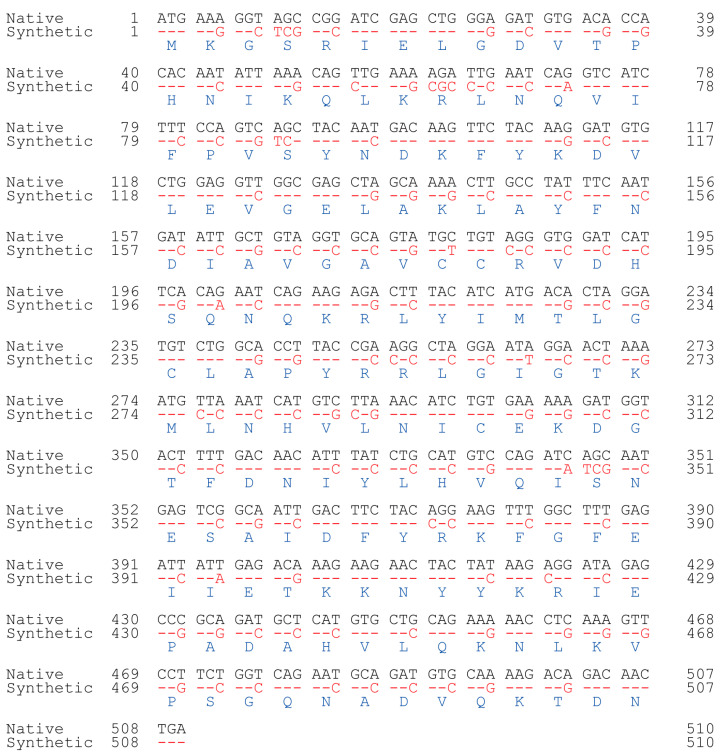
Nucleotide alignment of native (black; BAB14397) and synthetic (red) human *Naa50*. Identity is denoted by red dashes. Blue, amino acids.

A nonredundant search of the NCBI and NIH protein sequence databases (http://www.ncbi.nlm.nih.gov/, accessed on 7 November 2019) using BLASTp revealed that the archaeal SNAT protein [10] had ~20% homology to the human *N*-alpha-acetyltransferase50 (Naa50) gene and ~15% homology to the *N*-alpha-acetyltransferase60 gene (Figure 1A). We selected human Naa50 (also named NatE.; 169 amino acids) because it has higher homology to the archaeal SNAT than human Naa60. In addition, human Naa60 (also named NatF.; 249 amino acids) is exclusively localized to the golgi apparatus and is considerably larger than the archaeal SNAT (151 amino acids). By contrast, human Naa50 localizes to the cytoplasm [25], as does human SNAT [26]. The full-length human Naa50 was synthesized based on the amino acid sequence information in GenBank (BAB14379) and the codon usage of rice *SNAT2* (GenBank accession no. AK068156). Because rice *SNAT2* has a high G+C content (70%), we increased the G+C content from 41% (native hNaa50) to 59% (synthetic hNaa50) (Figure 2). Among the 170 codons in hNaa50, 99 were modified in synthetic hNaa50, in which the third nucleotide position in a codon was frequently changed from A or T to G or C, increasing the G+C content (Figure 1B and Figure 2).

### 3.2. Serotonin N-Acetyltransferase Enzyme Activity and Kinetics of hNaa50

**Figure 3:**
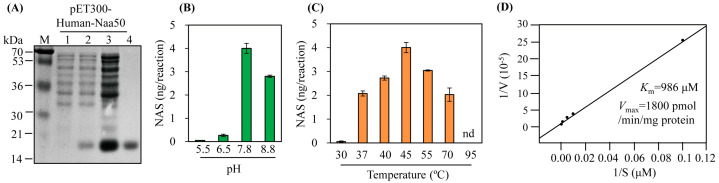
(**A**) Purification of N-terminal His × 6-tagged human Naa50. Serotonin *N*-acetyltransferase activity (conversion of serotonin into *N*-acetylserotonin [NAS]) as a function of (**B**) pH, (**C**) temperature, and (**D**) *K*_m_ and *V*_max_ values of human Naa50. *E*. *coli* BL21 (DE3) cells harboring the pET300-human Naa50 plasmid were induced with isopropyl *β*-D-thiogalactopyranoside (IPTG) for 5 h at 28 °C. M, molecular mass standards; lane 1, total proteins in 15 µL aliquots of bacterial culture without IPTG.; lane 2, total proteins in 15 µL aliquots of bacterial culture with IPTG.; lane 3, 20 µg soluble protein; lane 4, 5 µg protein purified by affinity chromatography. Protein samples were separated via 12% SDS-PAGE and stained with Coomassie blue. SNAT activity was measured based on *N*-acetylserotonin production in the presence of 0.5 mM serotonin at 45 °C and pH 7.8. *K*_m_ and *V*_max_ values were determined using Lineweaver-Burk plots. In vitro enzymatic products were measured via high-performance liquid chromatography (HPLC). Values are means ± SDs (*n* = 3).

The synthetic *hNaa50* gene was expressed as a hexahistidine fusion protein followed by Ni^2+^ affinity purification (Figure 3A). Purified recombinant hNaa50 was assayed for catalytic activity (conversion of serotonin to *N*-acetylserotonin); its SNAT activity was 43 pkat/mg protein. To investigate the SNAT kinetics of hNaa50, we carried out optimum pH and temperature experiments. As shown in Figure 3B, the highest SNAT activity was at pH 7.8, lower than many plant SNAT proteins [17,18,27,28], but higher than SNAT proteins from animals (optimum pH 6.7) [29,30]. In addition, the optimum pH for *N*-alpha-acetyltransferase activity of hNaa50 was 7.5, consistent with its SNAT activity [31]. hNaa50 exhibited peak activity at 45 °C followed by 55 °C, 40 °C, and 37 °C. Interestingly, hNaa50 showed SNAT activity at a level nearly 50% of the maximum level at 70 °C, as plant SNAT proteins do [32], whereas hNaa50 had an optimum temperature of 37 °C for *N*-alpha-acetyltransferase activity [31]. The *K*_m_ and *V*_max_ values for SNAT activity were 986 μM and 1800 pmol/min/mg protein, respectively. The *K*_m_ value of hNaa50 for serotonin was similar to that of human SNAT protein (*K*_m_ 1235 µM) [30], but higher than those of plant SNAT proteins [32]. Therefore, hNaa50 likely catalyzes the conversion of serotonin into *N*-acetylserotonin, followed by melatonin synthesis in human cells. Its involvement in melatonin biosynthesis in humans warrants further investigation.

### 3.3. Subcellular Localization of hNaa50 in Tobacco Leaves

**Figure 4:**
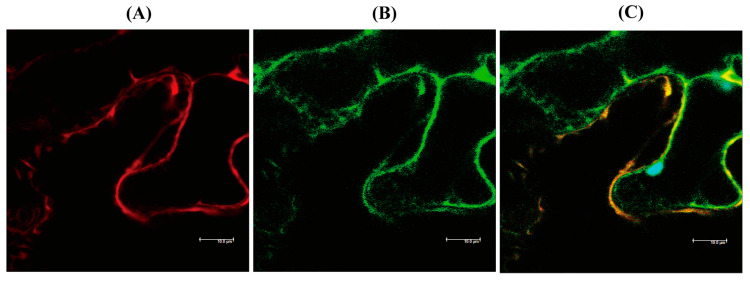
Localization of human Naa50. (**A**) Red fluorescence of hNaa50-mCherry. (**B**) Green fluorescence of cytoplasmic GFP. (**C**) Merged image (**A**+**B**). Leaves of 30-day-old tobacco (*Nicotiana benthamiana*; native Australian species) leaves were infiltrated with *Agrobacterium* (GV2260) containing XVE-inducible hNaa50-mCherry, or constitutive 35S:GFP (cytosolic marker). Bars, 10 µm.

hNaa50 localizes to the cytoplasm in human cells [25]. To determine whether hNaa50 is expressed in the cytoplasm in plants, the hNaa50-mCherry fusion protein was transiently expressed in tobacco leaves, and its localization was investigated by confocal microscopy. As shown in Figure 4, hNaa50-mCherry was expressed in the cytoplasm in tobacco leaves, as indicated by the overlapped expression of the cytoplasmic marker green fluorescent protein (GFP). Therefore, hNaa50 is expressed in the cytoplasm in plants and animals alike.

### 3.4. Characterization of Transgenic Rice Plants Overexpressing hNaa50

**Figure 5:**
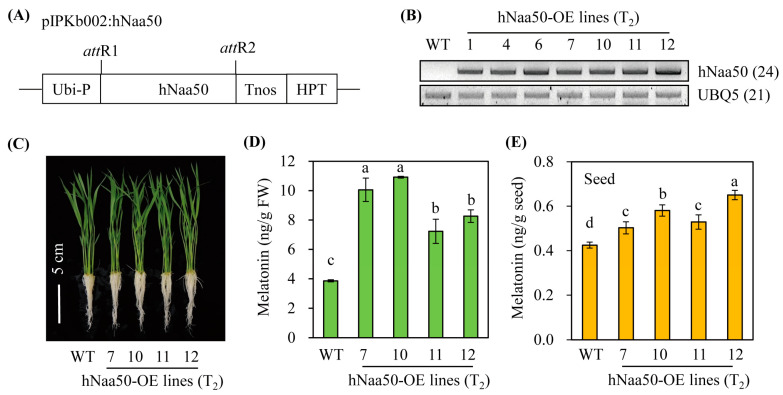
(**A**) Schematic diagram of a binary vector designed for *hNaa50* overexpression. (**B**) Reverse transcription (RT)-polymerase chain reaction (PCR) analyses of transgenic and wild-type rice plants from 7-day-old rice seedlings. (**C**) Phenotypes of 7-day-old seedlings. (**D**) Melatonin contents in 7-day-old rice seedlings challenged with 100 µM of 5-methoxytrymtamine for 24 h. (**E**) Seed melatonin contents. *Ubi-P*, maize ubiquitin promoter; *HPT*, hygromycin phosphotransferase; *Tnos*, nopaline synthase terminator; *attR1* and *attR2*, recombination sites; WT, wild type; *UBQ5*, rice ubiquitin 5 gene; *hNaa50*, human Naa50. The GenBank accession numbers of *hNaa50* and *UBQ5* are BAB14397 and Os03g13170, respectively. Different letters indicate significant differences (Tukey’s honestly significant difference test; *p* < 0.05).

To gain insight into the role of *hNaa50* in melatonin biosynthesis in vivo, we generated transgenic rice plants overexpressing *hNaa50* under the control of the maize ubiquitin promoter (Figure 5A). Of 12 independent T_1_ transgenic rice lines, seven independent lines showing a 3:1 hygromycin segregation ratio, indicative of a single copy insertion in the rice genome, were selfed to generate T_2_ homozygous seeds. Seven homozygous transgenic rice plants overexpressing *hNaa50* (*hNaa50*-OE) were monitored for the overexpression of the *hNaa50* transcript. As shown in Figure 5B, *hNaa50*-OE plants showed ectopic overexpression of *hNaa50* transgenes, as evidenced by RT-PCR, whereas no *hNaa50* transcript was found in the wild type. Melatonin levels were significantly elevated in *hNaa50*-OE compared to wild type when 7-day-old rice seedlings were rhizospherically treated with 100 µM of 5-methoxytryptamine, a precursor of melatonin biosynthesis by SNAT (Figure 5C,D) [2]. Moreover, seeds of the *hNaa50*-OE lines produced more melatonin than the wild type (Figure 5E). Collectively, these data indicate that ectopic overexpression of *hNaa50* is functionally coupled with melatonin biosynthesis in rice plants.

### 3.5. hNaa50-OE Rice Exhibit Osmotic Stress Tolerance

**Figure 6:**
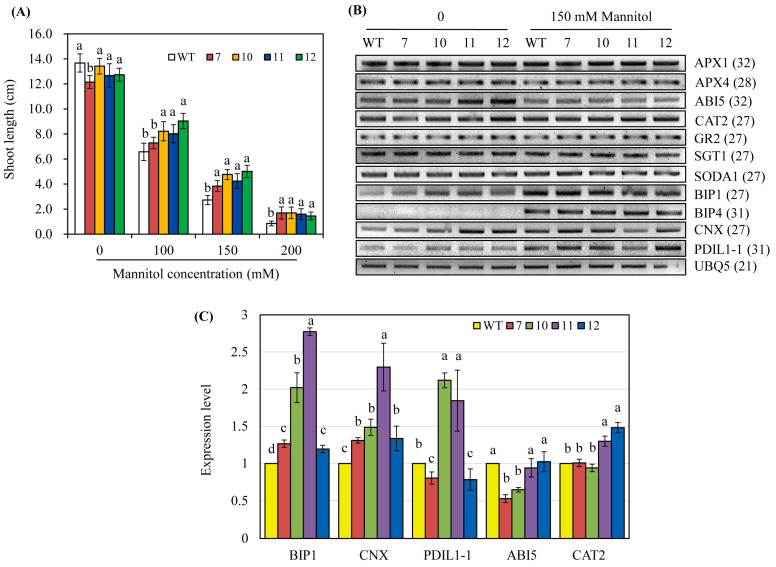
(**A**) Shoot length in the presence of mannitol. (**B**) Expression levels of genes involved in antioxidant and chaperone systems under non-stress and mannitol stress conditions. (**C**) Quantitative real-time polymerase chain reaction (qRT-PCR) analyses under non-stress condition. GenBank accession numbers: *APX1*, ascorbate peroxidase 1 (AB050724); *APX4*, (Os08g0549100); *ABI5*, abscisic acid insensitive 5 (Os01g0859300); *CAT2*, catalase 2 (Os02g0115700); *GR2*, glutathione reductase 2 (Os02g0813500); *SGT1*, suppressor of the G2 allele of skp1 (Os01g0624500); *SODA1*, superoxide dismutase A1 (Os05g0323900); *BIP1*, binding immunoglobulin protein 1 (AK119653); *BIP4* (AK106696); *CNX*, calnexin (AK069118); *PDIL1–1*, protein disulfide isomerase like 1–1 (AK068268); *UBQ5* (Os03g13170). Different letters indicate significant differences (Tukey’s honestly significant difference test, *p* < 0.05).

Melatonin is involved in tolerance to biotic and abiotic stresses [5,24,33,34]. Surface-sterilized seeds of the wild-type and *hNaa50*-OE lines were sown in a half-strength MS medium containing various concentrations of mannitol and grown for 8 days under light. The *hNaa50*-OE lines grew better than the wild type; shoot length was longer in the *hNaa50*-OE lines than the wild type, particularly at 150 and 200 mM mannitol concentrations (Figure 6A). This indicated that the increased melatonin level in *hNaa50*-OE confers tolerance to osmotic stress. As shown in Figure 6B, the antioxidant system-related genes, including ascorbate peroxidases (*APX1* and *APX4*), catalase 2 (*CAT2*), glutathione reductase 2 (*GR2*), superoxide dismutase A1 (*SODA1*), suppressor of the G2 allele of skp1 (*SGT1*), and abscisic acid (ABA)-associated signaling genes such as ABA insensitive 5 (*ABI5*), were altered in the *hNaa50*-OE lines compared to the wild type in the absence of mannitol. Of note, chaperone-related genes such as binding immunoglobulin protein 1 (*BIP1*) and calnexin (*CNX*) were markedly upregulated in the *hNaa50*-OE lines compared to the wild type under non-stress condition (Figure 6C). Other chaperone-related genes, including *BIP4* and protein disulfide isomerase like 1–1 (*PDIL1–1*), were not differentially expressed in the *hNaa50*-OE lines. Therefore, protein quality control in the endoplasmic reticulum (ER) is important in melatonin-induced osmotic stress tolerance because *BIP1* and *CNX* are major chaperones in the ER [35].

## 4. Discussion

It is now known that archaea have the *SNAT* gene, which catalyzes the conversion of serotonin into *N*-acetylserotonin or 5-MT into melatonin, suggesting melatonin synthesis in archaea [10]. However, there are no reports on the presence of melatonin and its function in archaea. The first archaeal *SNAT* from *T*. *volcanium* was previously annotated as a TvArd1 (arrest-defective-1) with protein N-terminal acetyltransferase (NAT) activity, which transfers an acetyl group from acetyl coenzyme A to the N-terminus of various proteins [25,36,37].

Plant SNAT enzymes show *N*-acetyltransferase activity towards histone [14] and chloroplast proteins [15], indicative of the broad substrate specificity of SNAT enzymes [2]. Thus, it is tempting to speculate that human Naa50, the closest homolog of archaeal SNAT, may harbor SNAT in addition to NAT activity. As expected, human Naa50 acetylated serotonin into *N*-acetylserotonin in vitro, indicating a bifunctional enzyme toward proteins and serotonin, as do plant SNAT enzymes [2]. However, the in vivo SNAT function of human Naa50 was unclear. Given the key role played by *SNAT* in melatonin biosynthesis, our finding of an archaeal *SNAT* ortholog gene in humans triggers a new possibility for the role of *Naa50* in melatonin biosynthesis in conjunction with the previously reported human *SNAT* [6]. In humans, it is known that *SNAT* mRNA expresses in certain tissues such as the pineal gland and retina, whereas other tissues including the heart, skin, and gastrointestinal tract have no such *SNAT* expression [6]. In contrast, *Naa50* expresses in all cells because Naa50 serves as the catalytic component of larger complexes of N-acetyltransferase to acetylate substrate proteins in a cotranslational manner [25]. Thus, it is tempting, by way of extrapolation, to presume that *Naa50* may be responsible for the synthesis of melatonin in human cells where *SNAT* does not express.

To evaluate the in vivo activity of human Naa50 in melatonin biosynthesis, we introduced *hNaa50* into the rice genome, generating transgenic rice plants stably overexpressing *hNaa50* (*hNaa50*-OE). Rice is a model system to study melatonin biosynthesis because rice not only contains all genes for melatonin biosynthesis but also produces relatively high levels of melatonin compared to other plants, including *Arabidopsis thaliana* and cassava [2]. These genetic and biochemical advantages of rice could provide a good biological system to easily evaluate the effects of ectopic overexpression of melatonin biosynthetic genes [38]. *hNaa50*-OE plants produced more melatonin than the wild type, indicating that human Naa50 is functionally coupled with melatonin biosynthesis in plant cells (Figure 5). Many transgenic plants overexpressing *SNAT* genes from plants or animals have been generated to overproduce melatonin [38,39,40,41]. These *SNAT*-OE transgenic plants have tolerance to biotic and abiotic stresses, including pathogens [42], salt [43], drought [40], cadmium [44,45], UV-B [46], and cold [39]. This is ascribed to the antioxidant activity of melatonin [34,47,48,49,50] and its induction of the expression of a range of defense genes [35,51,52]. In particular, under non-stress conditions, melatonin enhances growth and yield by increasing photosynthesis [52,53] and modulating plant-growth hormones [54,55]. The absolute level of melatonin throughout the plant life cycle affects the biosynthesis of gibberellic acids [18], brassinosteroids [56,57], and cytokinins [58], influencing flowering and grain yield. Indeed, the *hNaa50*-OE rice plants showed greater melatonin synthesis than the wild type, enhancing tolerance to osmotic stress (Figure 6). The enhanced osmotic stress tolerance in *hNaa50*-OE rice plants is ascribed to the enhanced protein quality control caused by increased melatonin production, as indicated by the upregulation of ER chaperone genes such as *BIP1* and *CNX*. Maintenance of ER activities in the presence of stress is important for stress tolerance because more than one-third of plant proteins are post-translationally modified in the ER [59]. BIP, as a heat shock protein 70-like chaperone protein, binds various nascent proteins to prevent their aggregation in the presence of stresses [59]. *Arabidopsis* overexpressing the pepper gene *BIP1* exhibits enhanced tolerance to osmotic and drought stress [60]. In addition, the ER molecular chaperone calnexin (CNX) directly binds many nascent proteins and assists their folding [59]. Rice *CNX* overexpression in tobacco enhances drought and cold tolerance [61]. Based on the roles of melatonin in plant protein quality control [35], melatonin-induced overexpression of chaperone genes such as *BIP1* and *CNX* in *hNaa50*-OE plants may enhance tolerance to osmotic stresses such as mannitol. The responses against other stresses, including salt and cadmium, in the *hNaa50*-OE plants were comparable to those of the wild type (data not shown). Similar to our results, an Arabidopsis knockout mutant of *Naa50* exhibited mannitol hypersensitivity [62].

The mechanisms through which human Naa50 mediates melatonin biosynthesis warrant further investigation. In addition, overexpression of *TvSNAT* homologs will likely show them to have other functions in fungi, animals, and plants [63,64].

## 5. Conclusions

We previously reported the cloning of serotonin *N*-acetyltransferase (*SNAT*) in the archaeon *Thermoplasma volcanium* (*TvSNAT*) [10]. In this study, we identified its ortholog in humans, an *N*-alpha-acetyltransferase50 (*Naa50*) with about 20% amino acid homology to TvSNAT. To determine whether human *Naa50* (*hNaa50*) shows SNAT activity, the full-length nucleotides of *hNaa50* were chemically synthesized and expressed in *Escherichia coli,* and the SNAT activity of the purified recombinant protein was measured. Recombinantly purified hNaa50 exhibited SNAT enzyme activity. The *K*_m_ and *V*_max_ values of hNaa50 toward serotonin were 986 μM and 1800 pmol/min/mg protein, respectively. Confocal microscopy revealed that hNaa50 is expressed in the cytosol of tobacco leaves. To investigate whether *hNaa50* is functionally coupled to melatonin biosynthesis in vivo, we generated transgenic rice plants overexpressing *hNaa50*. Those transgenic rice plants produced more melatonin than wild-type plants and showed enhanced osmotic stress tolerance due to the overproduction of melatonin. To the best of our knowledge, this is the first report that *hNaa50* encodes SNAT activity in addition to *N*-alpha-acetyltransferase activity. This suggests the presence of an alternative melatonin synthesis pathway in humans. Although it is unclear whether *hNaa50* is functionally linked to melatonin synthesis in human cells, we have opened a wide range of possibilities for *hNaa50* in melatonin biosynthesis in animal areas.

## Data Availability

The data presented in this study are available within the article.

## Supplementary Materials

The following supporting information can be downloaded at: https://www.mdpi.com/article/10.3390/antiox12020319/s1, Table S1: Sequences of primers used for polymerase chain reaction.
