# Supplementary material for: Human Naa50 Shows Serotonin N-Acetyltransferase Activity, and Its Overexpression Enhances Melatonin Biosynthesis, Resulting in Osmotic Stress Tolerance in Rice
Source: Antioxidants (Basel). 2023 Jan 30;12(2):319. doi: 10.3390/antiox12020319 (PMC9952165; doi:10.3390/antiox12020319)
Supplement: Supplementary file 1 [file antioxidants-12-00319-s001.zip › antioxidants-2193520-supplementary.pdf]

Supplemental Table S1. Sequences of primers used for polymerase chain reaction.

Gene	Forward (5´-3´)	Reverse (5´-3´)
hNaa50	GGCGCGCCATGAAGGGCTCGCGCATC	GGCGCGCCGGTTGTCCGTCTTCTGGAC
APX1	CAA GGA GGA GAT ACC CAC CA	TAG GTG GTC AGA ACC CTT GG
APX4	TGC ATC TAC GAA ATC TG	CTT CTT AGA AGC CTC
ABI5	GAT CAT TTT CCT TGC CGC TAC	CAA GTG TCA TCT CAC CTA GTG T
CAT2	CCT ATG CTG ATA CCC AAA G	CAC ACT GCG ACC AGT AGG A
GR2	TTA TCC AGG GTA TGG C	TGG CTT GGA TGA TG
SGT1	TAC AGG CAC GAC TTC T	GTA CGG CTC CTC TCC A
SODA1	ATT CGA TCT TCT GGA ATA ACC	TGC GTA TTT CCA GTT CAT CA
BIP1	TGA ACG TGA AGG CTG AGG AC	GTA GGT CTC GAG CTG GTT GC
BIP4	CAA GGA GGA GTA CGA GGA GAA G	CAC ACT TTC GAT CGA ATC CAA AC
CNX	CGC CGG AGG TCC CGA AGG GAG ACA	CTA GCT TGC ACT GAA CCT CAC AC
PDIC1-1	ACA AGT GAG TAG GAG AGG GCA TGG	CAA CTT GTC CAG AAC CAC TTC TCT A
UBQ5	CCG ACT ACA ACA TCC AGA AGG AG	AAC AGG AGC CTA CGC CTA AGC
